# Antibacterial activity of plant species used for oral health against *Porphyromonas gingivalis*

**DOI:** 10.1371/journal.pone.0239316

**Published:** 2020-10-08

**Authors:** Danielle H. Carrol, François Chassagne, Micah Dettweiler, Cassandra L. Quave

**Affiliations:** 1 Center for the Study of Human Health, Emory University College of Arts and Sciences, Atlanta, GA, United States of America; 2 Department of Dermatology, Emory University School of Medicine, Atlanta, GA, United States of America; University of the Pacific, UNITED STATES

## Abstract

*Porphyromonas gingivalis* is the keystone pathogen of periodontitis, a chronic inflammatory disease which causes tooth loss and deterioration of gingiva. Medicinal plants have been traditionally used for oral hygiene and health and might play a role as antibacterial agents against oral pathogens. In this work, we aimed to evaluate the antibacterial activity of plants used for oral hygiene or symptoms of periodontitis against *P*. *gingivalis*. We first reviewed the literature to identify plant species used for oral hygiene or symptoms of periodontitis. Then, we cross-checked this species list with our in-house library of plant extracts to select extracts for testing. Antibacterial activity tests were then performed for each plant extract against *P*. *gingivalis*, and their cytotoxicity was assessed on HaCaT cells. The selectivity index (SI) was then calculated. A total of 416 plant species belonging to 110 families and 305 genera were documented through our literature search, and 158 plant species were noted as being used by North American Native peoples Once cross-checked with the extracts contained in our library of natural products, 30 matches were identified and 21 were defined as high priority. Of the 109 extracts from 21 plant species selected and tested, 21 extracts from 11 plants had higher than 90% inhibition on *P*. *gingivalis* at 64 μg/mL and were further selected for MIC (Minimum Inhibitory Concentration) assays. Out of 21 plant extracts, 13 extracts (7 plant species) had a SI > 10. *Pistacia lentiscus* fruits showed the best MIC with value of 8 μg/mL, followed by *Zanthoxylum armatum* fruits/seeds with a MIC of 16 μg/mL. *P*. *lentiscus* fruits also showed the highest SI of 256. Most of the extracts tested present promising antibacterial activity and low cytotoxicity. Further testing for biofilm eradication and examination of activity against other dental pathogens and oral commensals should be performed to confirm the potential of these extracts as antibacterial agents. Future work will focus on application of a bioassay-guided fractionation approach to isolating and identifying the most active natural products in the top performing extracts. This study can serve as a basis for their future development as ingredients for oral hygiene products.

## Introduction

### *Porphyromonas gingivalis*, periodontitis, and other chronic inflammatory diseases

Periodontitis, a chronic inflammatory disease which causes tooth loss and deterioration of gingiva, alveolar bone, and periodontal ligaments, is caused by several microbes including the keystone pathogen *Porphyromonas gingivalis* [1]. This bacterium is a gram negative, rod-shaped, obligate anaerobe belonging to the 500 bacterial species living in the oral cavity [2]. It infects periodontal tissues as a secondary infection through interactions with commensal streptococci [3]. Because of its ability to evade the host immune response and travel from cell to cell as well as via the haematogenous route, *P*. *gingivalis* can cause and maintain high levels of chronic inflammation in various peripheral organs [4]. Infection with *P*. *gingivalis* has been associated with cardiovascular disease, diabetes mellitus, respiratory infection, rheumatoid arthritis, osteoporosis, obesity, and preterm birth [5]. While potential mechanisms linking periodontal infection with these disease states are not fully understood, several models have been hypothesized and explored, reviewed by Kim and Amar [5]. More recently, the link between inflammation and the central nervous system (CNS) has been receiving increasing attention, and many studies have begun to investigate the link between *P*. *gingivalis*, inflammation, and the brain. Although the exact cause and effect relationship between this bacteria, peripheral and CNS inflammation, and the brain has yet to be fully elucidated, an increasing volume of clinical and experimental evidence has associated *P*. *gingivalis* with Alzheimer’s disease (AD), dementia, and other forms of cognitive decline [1, 6–9].

### *P*. *gingivalis* resistance and treatment

Currently, periodontitis is treated with systemic and local antibiotics and occasionally surgery to reach deep-pocket inflammation [10]. Antibiotics are used to target anaerobic bacteria, especially *P*. *gingivalis*. A variety of antibiotics are employed, including metronidazole, amoxicillin, amoxicillin/clavulanic acid, clindamycin, tetracycline and fluoroquinolones [11, 12]. However, *P*. *gingivalis* strains show resistance levels as high as 21.56% to metronidazole, 25.49% to amoxicillin, and 23.52% to clindamycin [13]. Significant levels of resistance have also been recorded for penicillin, erythromycin, azithromycin, and tetracycline [14]. While antibiotic resistance is rising, new antimicrobials are increasingly necessary, but in recent years there has in fact been a decrease in the discovery of new antibiotics [15]. To counteract this lack of drug discovery, new treatments for *P*. *gingivalis* have been documented. This includes photo-activated disinfection, small molecule inhibitors, local drug delivery systems, and immune-based therapy [16–18].

### *P*. *gingivalis* and medicinal plants

Another solution for *P*. *gingivalis* infections could come from medicinal plants. Not only are plant species a rich source of antibacterials [19], but ethnobotanical information can guide the selection of plant extracts for discovery of new antibacterials [20, 21]. Indeed, there is a vast wealth of recorded ethnobotanical information concerning the use of plants for oral health. For example, clove oil (*Syzygium aromaticum* (L.) Merr. & L.M. Perry, Myrtaceae) is widely used for managing dental pain; the twigs of miswak (*Salvadora persica* L., Salvadoraceae) are used as a toothbrush in the Middle East and Africa, and neem twigs (*Azadirachta indica* L., Meliaceae) are employed as oral deodorant, toothache reliever, tongue cleaner and toothbrush in Asia [22–24]. While medicinal plants are widely used for various oral health conditions, more research is needed to link these ethnomedicinal uses to pharmacological activity on dental pathogens. Previous studies of medicinal plant extracts against *P*. *gingivalis* have found antimicrobial activity, but often only at high concentrations; for example, a *Citrus sinensis* peel extract and an extract of *Camellia sinensis* were both found to have MICs (minimum inhibitory concentrations) of 12.5 mg/mL [25–29].

### Objectives

The objective of this study was to identify plant species traditionally used in the treatment of dental disorders with growth inhibitory activity and good selectivity against *Porphyromonas gingivalis*. This approach was based on the rationale that *P*. *gingivalis* is linked to dental diseases (especially periodontitis) and ethnobotanical data on the long-standing use of plants for oral hygiene and in the treatment of dental disorders could serve as a guide to identifying natural products with activity against this pathogen.

## Materials and methods

### Ethnopharmacological selection of plants

We performed a literature search by reviewing books related to medical botany and Native American plants, articles published in the *Journal of Ethnopharmacology*, and scientific articles found in the PubMed database. This literature search was not a comprehensive systematic review. We sought plants that have been used for oral hygiene or for symptoms of periodontitis including toothache, sore mouth, mouth abscesses, loose teeth, and halitosis. Specific keywords were used to search the online databases, such as “medicinal plant,” combined with terms related to dental disorders, any symptoms of periodontitis, and oral hygiene/health, such as “oral health,” “oral hygiene,” “dental disorders,” “toothache,” “halitosis,” “sore throat.” For each plant species, we recorded information on plant family, genus, species, part of plant used, medical systems, and application. After compiling these sources, all reported plant names were cross-checked for accuracy with The Plant List (http://www.theplantlist.org/), and any botanical synonyms or citations with unaccepted author epithets were updated to the current corrected nomenclature. Then, an additional literature search was performed to check whether the plant species had been previously tested against *Porphyromonas gingivalis*. The plant list created from this review was cross-checked with the extracts contained in the Quave Natural Product Library (QNPL), an in-house plant extract library developed in our laboratory. Matches were sorted according to priority level. Priority levels were defined as follows: 1) genus, species, and plant part match; 2) genus and species match, but different plant part; 3) genus and part match; 4) genus, species, and part match but the plant is part of a multi-ingredient medicine.

### Plant materials

The QNPL is a plant extract library composed of 2,000 extracts from 600 plant species belonging to 52 plant families. All plants have been collected under appropriate permits and with express permission from landowners. All of the extracts are stored in the phytochemistry laboratory of the Quave Research Group (Emory University, Atlanta, GA, US). A voucher specimen for each species is also deposited at the Emory University Herbarium (GEO). Various plant parts from the same plant species were extracted with different solvents. For aqueous extractions, samples were extracted in Type II distilled water at a ratio of 1 g plant material:10 mL H_2_O and boiled for 20 minutes. The extraction products were then filtered after cooling at room temperature, concentrated using a rotary evaporator (Buchi®, Flawil, Switzerland), and then shell-frozen and lyophilized for 24 hours. For methanol and ethanol extractions, plant materials were mixed at a ratio of 1 g:10 mL with 80% methanol and 80 or 95% ethanol respectively for 72 hours under constant agitation. This step was repeated one time using the same plant residue, and then both extraction products were filtered and combined. The alcoholic filtrate was concentrated using a rotary evaporator, shell-frozen and lyophilized for 24 hours. All extracts were stored dry at -20°C before being dissolved in 100% dimethyl sulfoxide (DMSO) at a stock concentration of 10 mg/mL for the assays.

### Bacterial strains and growth conditions

Freeze dried *P*. *gingivalis* strain ATCC® 33277 was rehydrated in Brain Heart Infusion (BHI) media supplemented with 5 μg/mL hemin (Alfa Caesar®, Heysham, UK) and 1 μg/mL menadione (Alfa Caesar®, Heysham, UK). Bacteria were grown on supplemented BHI (sBHI) agar plates (5 μg/mL hemin, 1 μg/mL menadione) and supplemented blood agar plates (5% defibrinated sheep’s blood (Hemostat Laboratories, Dixon, CA), 5 μg/mL hemin, 1 μg/mL menadione, 2 g/L yeast extract) [30–32]. Hemin stock was prepared by dissolving 250 mg hemin in 5 mL of 1M NaOH and 495 mL deionized distilled water (ddH_2_O) for a final concentration of 0.5 mg/mL. Menadione stock was prepared by dissolving 25 mg menadione in 20 mL 100% ethanol for a final concentration of 1.25 mg/mL. Each stock was filter sterilized, wrapped in aluminum foil, and stored at 4°C.

For MIC assays, sBHI was pre-incubated at 37°C for 24 hours before the bacterial culture. The bacterial culture was incubated in the prereduced sBHI for 48 hours in anaerobic conditions at 37°C (BD GasPak® container system, Franklin Lake, NJ). Additional sBHI was pre-incubated 24 hours before the assay [30].

### Growth inhibition assays

The minimum inhibitory concentration (MIC) was defined as the lowest concentration at which 90% of growth was inhibited (corresponding to the lowest concentration of no visible growth in the well) compared with vehicle control, as previously reported [33, 34], and the IC_50_ was defined as the lowest concentration at which 50% of growth was inhibited. As there is no standard method described in the CLSI (Clinical and Laboratory Standards Institute) guidelines for growing and evaluating the MIC of *P*. *gingivalis*, we followed previously described methods with some modifications [30–32]. Briefly, the bacterial culture previously incubated for 48 hours was standardized to a final concentration of 10^6^ CFU/mL in sBHI using a Cytation 3 multimode plate reader (Biotek®, Winooski, VT) by change in optical density (OD_600_ nm), and confirmed by colony plate counts. Assays with plant extracts and controls were performed in 96-well flat-bottom non-tissue culture treated plates (Falcon® 35–3075, Corning, NY). All extracts were tested at a range concentration of 2–256 μg/mL or 1–128 μg/mL via serial dilution. An untreated growth control, vehicle (DMSO) control (S1 Fig), and antibiotic control (tetracycline) were included. Plates were incubated in anaerobic conditions at 37°C for 72 hours. All experiments were performed in triplicate and repeated one time on a separate day to confirm the accuracy of the results. Growth inhibition was determined by change in OD from the start of incubation to the final time point (72 hours). Growth inhibition was calculated with the following formula: (1−(Δ*ODtest*/Δ*ODvehicle*))*100 [35]. The mean and standard error of triplicates for each treatment were calculated using Microsoft Excel.

### Cytotoxicity assays

Human immortalized keratinocytes (HaCaTs) were used to evaluate the cytotoxic activity of plant extracts on human gingival cells. HaCaTs represent a suitable substitute for gingival keratinocytes and are often used as such because they can be easily grown and passaged indefinitely [36]. HaCaTs were cultured in Dulbecco's Modified Eagle Medium (DMEM) with L-glutamine and 4.5 g/L glucose (Corning®, Corning, NY), supplemented with 10% heat-inactivated fetal bovine serum (Seradigm®, Randor, PA) and a 1X solution of 100 IU penicillin and 100 μg/mL streptomycin (Corning®, Corning, NY). The Lactate Dehydrogenase (LDH) assay was used to determine the cytotoxicity activity following manufacturer’s instructions (LDH assay kit, G-Biosciences, St. Louis, MO). Briefly, HaCaT cells were first standardized to a concentration of 4 x 10^4^ cells/mL using a hemocytometer, and then incubated for 48 hours (5% CO_2_) in 96-well flat-bottom tissue culture treated plates (Falcon®, Corning, NY). Then, treatments were added to HaCaT cells at a concentration range of 4–512 μg/mL or 1–128 μg/mL via serial dilution and incubated for 24 hours. Cells were then processed, and OD was measured at 490nm to determine the proportion of lysed cells. Cytotoxicity was calculated using the following formula: (*ODtest* − *OD_spontaneous_*)/(*OD_max_*)*100, where OD_max_ is the OD_490nm_ read for wells treated with lysis buffer after incubation to achieve maximum of cell lysis, and OD_spontaneous_ is the OD_490nm_ read for wells without treatment. Selectivity index (SI) was defined as the following: SI = (IC_50_ of extracts against human keratinocytes)/(IC_50_ of extracts against *P*. *gingivalis*).

## Results

### Selection of plants

**Fig 1:**
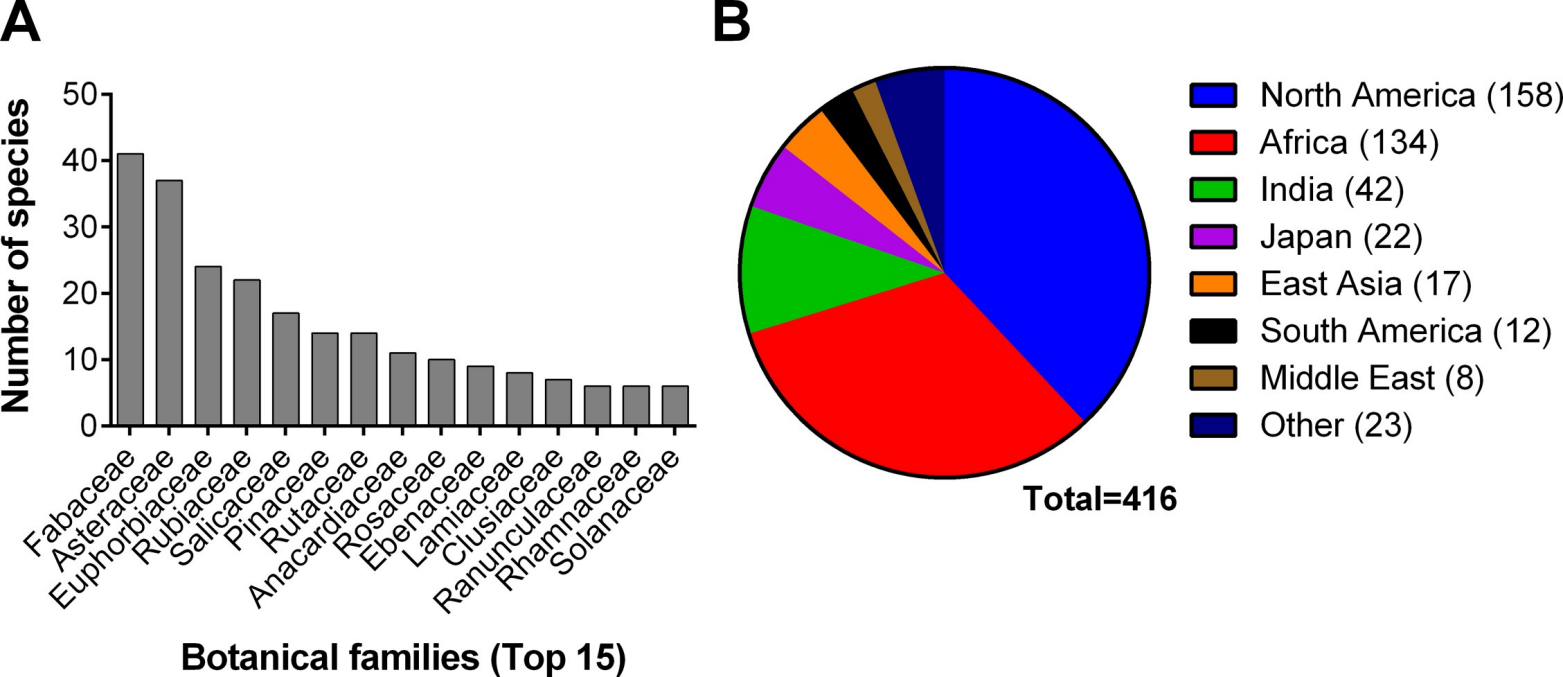
Most represented A) botanical families and B) medical systems by number of species in the literature review.

Our bibliographical review process covered a total of 16 publications including three books [37–39], and 13 scientific articles [32, 40–51]. One reference accounted for 81.4% of the 495 ethnobotanical uses recorded [37]. In total, 416 plant species belonging to 110 families and 305 genera were documented through our literature search. The most represented families were Fabaceae (41 plant species, 9.9%), Asteraceae (37 plant species, 8.9%), Euphorbiaceae (24 plant species, 5.8%) (Fig 1A). The most represented genera were *Salix* (9 plant species, 2.1%), *Diospyros* (7 plant species, 1.7%), and *Acacia* (6 plant species, 1.4%). One hundred seventy plant species were mentioned to be used as chewing sticks, 124 were used for periodontitis-like symptoms, 50 in cleaning gums, 32 for mouth sores, 25 for toothache, 17 for blackening/reddening, 13 as toothpaste, and 8 for halitosis. A total of 158 plant species were reported to be used in North America, 134 from Africa, 42 from India, and 22 from Japan (Fig 1B).

When cross-checked with the extracts contained in the QNPL, 30 matches were identified out of the 416 plant species recorded. Of the 30 matches, 21 plants were determined to be high priority (priority levels 1 and 2) and were selected for screening. All QNPL extracts from various parts of these plants and various extract solvents were selected, totaling 109 extracts.

### Growth inhibition assays

An initial screen was performed on 109 extracts from the 21 plant species found in the QNPL to select the most active extracts. All extracts which had higher than 90% inhibition on *P*. *gingivalis* at 256 μg/mL were defined as hits. The hits from this screen, 38 extracts from 17 plants, were chosen for a second screening at 64 μg/mL. The second screen yielded 21 extracts from 11 plants that were considered as hits (>90% inhibition) and were further selected for MIC assays by dose-response testing (S1 Fig). MIC values ranged from 8–128 μg/mL. *Pistacia lentiscus* fruits exhibited the best MIC with a value of 8 μg/mL, followed by *Zanthoxylum armatum* fruits/seeds with an MIC of 16 μg/mL. Ten plant extracts had an MIC of 32 μg/mL, and eight had an MIC of 64 μg/mL (Table 1).

**Table 1:** Effect of 109 plant extracts from 21 plant species on growth inhibition in *P*. *gingivalis* (ATCC® 33277).

Family	Species	Voucher Specimen Accession Number*	Collection site	Ethnobotanical use	Part	Extract Solvent^a^	MIC (μg/mL)^b^	IC_50_ (μg/mL)^c^
Altiginaceae	*Liquidambar styraciflua* L.	GEO20428, GEO20429	Atlanta, GA, USA	Used as chewing sticks and chewing gums by Native American people (Cherokee) [37, 43]	woody part	MeOH	ND	ND
		GEO20428, GEO20429	Atlanta, GA, USA		leaf	MeOH	(128–256)*	(128–256)*
		GEO20428, GEO20429	Atlanta, GA, USA		fruit, seed	MeOH	ND	ND
		GEO22852, GEO22853	Ichauway, GA, USA		root	80% EtOH(aq)	(128–256)*	64
		GEO22852, GEO22853	Ichauway, GA, USA		leaf	80% EtOH(aq)	ND	ND
Anacardiaceae	*Pistacia lentiscus* L.	GEO22045	Levanzo, Italy	Resin used for toothache, tooth disease,or gum inflammation in the Middle East and Kampo medicine [42, 52].	leaf	95% EtOH(aq)	(128–256)*	(128–256)*
		GEO22045	Levanzo, Italy		leaf	dH_2_O	64	64
		GEO22045	Levanzo, Italy		woody part	95% EtOH(aq)	32	16
		GEO22045	Levanzo, Italy		woody part	dH_2_O	ND	ND
		GEO22045	Levanzo, Italy		leaf	95% EtOH(aq)	ND	ND
		GEO22045	Levanzo, Italy		fruit	95% EtOH(aq)	8	2
Asteraceae	*Achillea millefolium* L.	GEO20072	Ginestra, Italy	Leaf and stem used by Native American people (Crow) as tea held in mouth for sore gums [38, 49]	inflorescence	EtOH	ND	ND
		GEO20072	Ginestra, Italy		leaf, stem	EtOH	ND	ND
		GEO20072	Ginestra, Italy		flower, leaf, stem	EtOH	ND	ND
		GEO20072	Ginestra, Italy		flower, leaf, stem	MeOH	ND	ND
		GEO20072	Ginestra, Italy		inflorescence	MeOH	ND	ND
		GEO20072	Ginestra, Italy		leaf, stem	MeOH	ND	ND
		GEO20232	Monte Vulture, Italy		leaf, stem	MeOH	ND	ND
Ebenaceae	*Diospyros virginiana* L.	GEO22867	Ichauway, GA, USA	Boiled bark decoction for sore mouth of babies in Eastern North America [37]	stem	80% EtOH(aq)	ND	ND
		GEO22867	Ichauway, GA, USA		leaf	80% EtOH(aq)	(128–256)*	(128–256)*
		GEO22867	Ichauway, GA, USA		leaf	80% EtOH(aq)	ND	ND
		GEO22867	Ichauway, GA, USA		immature fruit	80% EtOH(aq)	ND	ND
		GEO22867	Ichauway, GA, USA		woody stem	80% EtOH(aq)	ND	ND
Fabaceae	*Tamarindus indica* L.	TTALBR541	Maracas Valley, Trinidad	Twig used as a chewing stick in West Africa [37]	leaf	95% EtOH(aq)	ND	ND
	*Vicia faba* L.	CQ-103	Ginestra, Italy	Ground dried beans used for sore mouth in North America [37]	flower, leaf, root, stem	EtOH	32	32
		CQ-103	Ginestra, Italy		aerial parts	MeOH	32	32
Fagaceae	*Quercus alba* L.	GEO20338	Atlanta, GA, USA	Bark used as a decoction for sore mouth in North America [37]	bark	MeOH	ND	ND
		GEO20338	Atlanta, GA, USA		gall	MeOH	ND	ND
		GEO20338	Atlanta, GA, USA		leaf	MeOH	ND	ND
		GEO20338	Atlanta, GA, USA		bark	dH_2_O	ND	ND
		GEO20338	Atlanta, GA, USA		gall	dH_2_O	ND	ND
		GEO20338	Atlanta, GA, USA		woody part	MeOH	ND	ND
		GEO20338	Atlanta, GA, USA		woody part	dH_2_O	ND	ND
Juglandaceae	*Carya alba* (L.) Nutt. ex Elliott	GEO20433	Atlanta, GA, USA	Inner bark chewed and blew into mouth for sore mouth by Native American people (Cherokee) [39]	woody part	MeOH	32	32
		GEO20433	Atlanta, GA, USA		leaf	MeOH	ND	ND
		GEO20433	Atlanta, GA, USA		fruit	MeOH	32	16
		GEO20433	Atlanta, GA, USA		leaf	80% EtOH(aq)	ND	ND
		GEO20433	Atlanta, GA, USA		bark	80% EtOH(aq)	ND	ND
		GEO20433	Atlanta, GA, USA		woody stem	80% EtOH(aq)	(128–256)*	(128–256)*
	*Juglans regia* L.	GEO23774	Ginestra, Italy	Stem and bark used for teeth cleaning as chewing sticks in Pakistan and India [37, 43]	woody stem	EtOH	64	32
		GEO23774	Ginestra, Italy		woody stem	MeOH	64	32
		CQ-181	Ginestra, Italy		immature fruit	EtOH	64	32
		CQ-181	Ginestra, Italy		leaf	EtOH	ND	ND
		CQ-181	Ginestra, Italy		woody part	EtOH	32	16
		CQ-181	Ginestra, Italy		woody part	MeOH	ND	ND
		CQ-181	Ginestra, Italy		immature fruit	MeOH	32	16
		CQ-181	Ginestra, Italy		leaf	MeOH	ND	ND
Lauraceae	*Sassafras albidum* (Nutt.) Nees	GEO22810, GEO22811	Ichauway, GA, USA	Used as a chewing stick for teeth cleansing in North America (Appalachia and Ozarks region) [37, 43]	leaf	80% EtOH(aq)	(128–256)*	(128–256)*
		GEO22810, GEO22811	Ichauway, GA, USA		root	80% EtOH(aq)	ND	ND
		GEO22810, GEO22811	Ichauway, GA, USA		stem	80% EtOH(aq)	64	64
Meliaceae	*Azadirachta indica* A.Juss.	TTALBR531	Sangre Grande, Trinidad	Twig used as cleaning stick in India [43, 45, 53]. Powdered inner bark held in mouth for toothache in India [44]	leaf, woody stem	95% EtOH(aq)	64	64
Moraceae	*Artocarpus altilis* (Parkinson ex F.A.Zorn) Fosberg	TTALBR542	Maracas Valley, Trinidad	Latex used for mouth sores by Hawaiian [37]	leaf	95% EtOH(aq)	ND	ND
Myricaceae	*Morella cerifera* (L.) Small	GEO20950, GEO20951	Arcadia,FL, USA	Rook bark used for oral hygiene in the Southern U.S. [37]. Bark used as a powder and decoction to prevent dental decay in Florida [54]	leaf, flower	MeOH	128	64
		GEO21165, GEO21169	Arcadia,FL, USA		woody part	MeOH	(128–256)*	(128–256)*
		GEO21165, GEO21169	Arcadia,FL, USA		woody stem	dH_2_O	ND	ND
		GEO20950, GEO20951	Arcadia,FL, USA		branch, stem	MeOH	ND	ND
		GEO20950, GEO20951	Arcadia,FL, USA		bark	MeOH	ND	ND
		GEO20950, GEO20951	Arcadia,FL, USA		bark	dH_2_O	ND	ND
		GEO20950, GEO20951	Arcadia,FL, USA		branch, stem	dH_2_O	ND	ND
Oleaceae	*Olea europaea* L.	GEO20084	Ginestra, Italy	Twig used as chewing stick in the Middle East [37]	leaf	EtOH	(128–256)*	64
		GEO20084	Ginestra, Italy		woody part	MeOH	(128–256)*	(128–256)*
		GEO20084	Ginestra, Italy		leaf	MeOH	64	32
Polygonaceae	*Rumex crispus* L.	GEO20070	Ginestra, Italy	Powdered root used as a toothpaste in North America [37]	aerial part, fruit, leaf, stem	EtOH	(128–256)*	(128–256)*
		GEO20070	Ginestra, Italy		ND	MeOH	ND	ND
Polypodiaceae	*Pleopeltis polypodioides* (L.) E.G. Andrews & Windham	GEO21158, GEO21159	Arcadia, FL, USA	Frond used as mouthwash by Native American people (Houma) [37]	whole plant	MeOH	ND	ND
		GEO21158, GEO21159	Arcadia, FL, USA		whole plant	dH_2_O	ND	ND
Rutaceae	*Citrus sinensis* (L.) Osbeck	GEO21152	Nocatee, FL, USA	Peeled twig used as chewing sticks in West Africa [37]	fruit rind	MeOH	ND	ND
		GEO21152	Nocatee, FL, USA		woody part	MeOH	32	16
		GEO21152	Nocatee, FL, USA		woody part	MeoH	ND	ND
	*Zanthoxylum armatum* DC.	GEO22201	Baral, Islamic Republic of Pakistan	Wood and bark used as chewing sticks in india [37]	fruit, seed	95% EtOH(aq)	16	16
Salicaceae	*Salix nigra* Marshall	GEO21184, GEO21185	Myakka City, FL, USA	Used by North American people (Iriquois) for periodontitis like symptoms [37]	leaf	MeOH	ND	ND
		GEO21184, GEO21185	Myakka City, FL, USA		leaf	dH_2_O	ND	ND
		GEO21066, GEO21024	Myakka City, FL, USA		flower, fruit, leaf	MeOH	ND	ND
		GEO21066, GEO21024	Myakka City, FL, USA		branch	MeOH	ND	ND
		GEO21210, GEO21212	Arcadia, FL, USA		woody stem	MeOH	(128–256)*	(128–256)*
		GEO21210, GEO21212	Arcadia, FL, USA		woody stem	dH_2_O	ND	ND
		GEO21066, GEO21024	Myakka City, FL, USA		branch	dH_2_O	ND	ND
		GEO21066, GEO21024	Myakka City, FL, USA		flower, fruit, leaf	dH_2_O	ND	ND
		GEO21185, GEO21184	Myakka City, FL, USA		woody stem	MeOH	ND	ND
		GEO21210, GEO21212	Arcadia, FL, USA		leaf	MeOH	ND	ND
		GEO21210, GEO21212	Arcadia, FL, USA		bark	MeOH	ND	ND
		GEO21185, GEO21184	Myakka City, FL, USA		bark	MeOH	ND	ND
		GEO21066, GEO21024	Myakka City, FL, USA		bark	MeOH	ND	ND
		GEO22870, GEO22871	Ichauway, GA, USA		leaf	80% EtOH(aq)	(128–256)*	(128–256)*
		GEO22870, GEO22871	Ichauway, GA, USA		bark	80% EtOH(aq)	ND	ND
		GEO22870, GEO22871	Ichauway, GA, USA		root	80% EtOH(aq)	(128–256)*	(128–256)*
		GEO21066, GEO21024	Myakka City, FL, USA		bark	dH_2_O	ND	ND
		GEO21185, GEO21184	Myakka City, FL, USA		woody stem	dH_2_O	(128–256)*	(128–256)*
		GEO21210, GEO21212	Arcadia, FL, USA		bark	dH_2_O	(128–256)*	(128–256)*
Sapotaceae	*Sideroxylon celastrinum* (Kunth) T.D. Penn.	GEO21090, GEO21084	Arcadia, FL, USA	Outer bark mucilage used a cleaning gum by North American people (Kiowa) [37]	stem	MeOH	ND	ND
		GEO21090, GEO21084	Arcadia, FL, USA		leaf, stem	MeOH	ND	ND
		GEO21090, GEO21084	Arcadia, FL, USA		leaf, stem	dH_2_O	ND	ND
		GEO21090, GEO21084	Arcadia, FL, USA		stem	dH_2_O	ND	ND
	*Sideroxylon lanuginosum* Michx.	GEO22922, GEO22923	Ichauway, GA, USA	Outer bark mucilage used a cleaning gum by North American people (Kiowa) [37]	leaf	80% EtOH(aq)	ND	ND
		GEO22982, GEO22983	Ichauway, GA, USA		leaf	80% EtOH(aq)	ND	ND
		GEO22982, GEO22983	Ichauway, GA, USA		woody stem	80% EtOH(aq)	ND	ND
		GEO22982, GEO22983	Ichauway, GA, USA		bark	80% EtOH(aq)	(128–256)*	(128–256)*
Vitaceae	*Vitis rotundifolia* Michx.	GEO22924, GEO22925	Ichauway, GA, USA	Ashes of burnt branches used as a toothpaste in England [37]	leaf, stem	MeOH	ND	ND
		GEO22924, GEO22925	Ichauway, GA, USA		leaf	80% EtOH(aq)	ND	ND
		GEO22924, GEO22925	Ichauway, GA, USA		root	80% EtOH(aq)	ND	ND
		GEO22924, GEO22925	Ichauway, GA, USA		woody stem	80% EtOH(aq)	ND	ND
		GEO22924, GEO22925	Ichauway, GA, USA		immature fruit	80% EtOH(aq)	ND	ND
	*Vitis vinifera* L.	GEO20102	Ginestra, Italy	Ashes of burnt branches used as a toothpaste in England [37]	stem	EtOH	ND	ND
		GEO20102	Ginestra, Italy		fruit	EtOH	(128–256)*	(128–256)*
		GEO20102	Ginestra, Italy		leaf	EtOH	64	64
		GEO20102	Ginestra, Italy		fruit	MeOH	ND	ND
		GEO20102	Ginestra, Italy		leaf	MeOH	32	64
		GEO20102	Ginestra, Italy		stem	MeOH	32	32

^**a**^dH_2_O: Distilled water. EtOH: Ethanol. EtOH(aq): aqueous ethanol. MeOH: methanol.

^b^Only the MIC and IC_50_ for plant extracts with a growth inhibition > 90% at 64 μg/mL are shown. ND: Not Determined. (128–256)*: MIC was not determined but the extract has a growth inhibition >90% at 256 μg/mL but not at 64 μg/mL, meaning that the MIC could be either 128 or 256 μg/mL.

^c^(128–256)*: In cases where the IC_50_ was not detected for the dose-response studies beginning at 64 μg/mL, but the extract has a growth inhibition >90% at 256 μg/mL, the IC_50_ could be either 128 or 256 μg/mL. As a point of comparison, the MIC for tetracycline was 0.125 μg/mL and IC_50_ was 0.063 μg/mL.

*All accessions with GEO have been digitized and can be viewed online with the SERNEC portal (http://sernecportal.org/portal/). To access, click on “Search Collections”, only include a checkbox for the Emory University Herbarium and click on search. Next, enter only the numeric portion of the GEO accession ID (e.g., for GEO20102, enter “20102” under the Specimen Criteria Catalog Number box). Then, click “List Display” to view the record.

### Cytotoxicity

**Fig 2:**
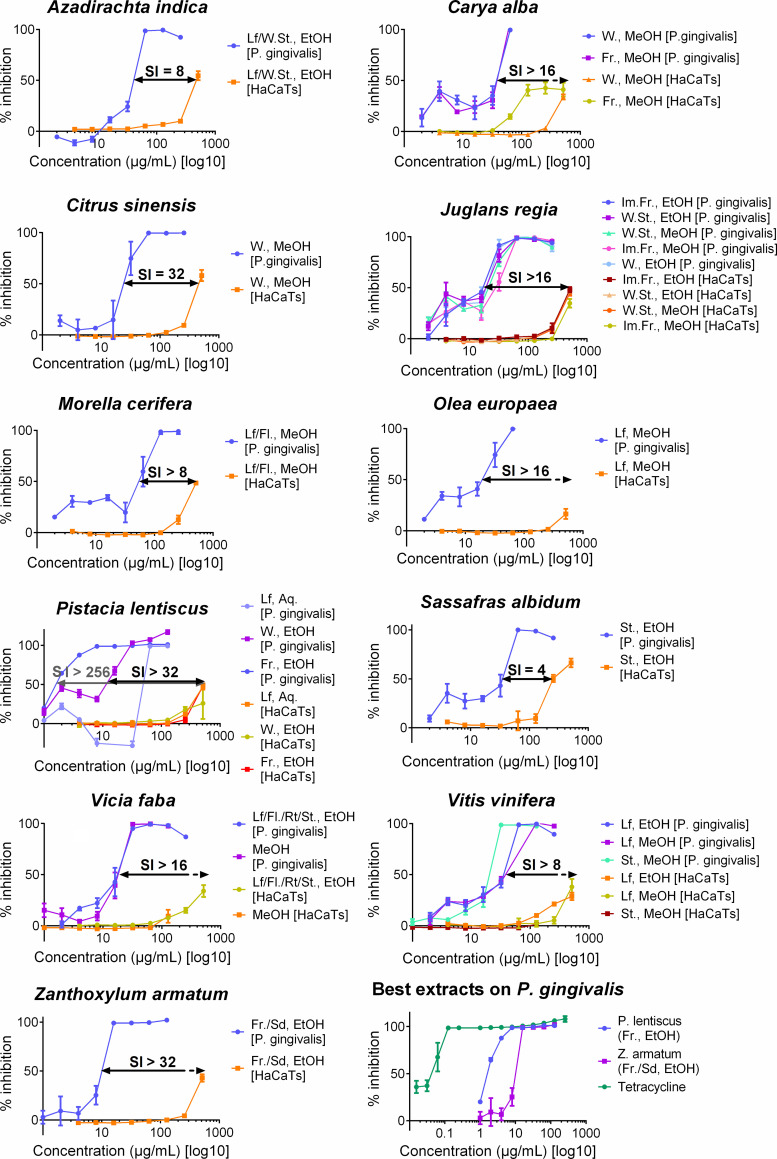
Dose response curves representing the growth inhibition on *Porphyromonas gingivalis* and the cytotoxicity on HaCaTs for the 11 plant species (21 extracts) tested. Twenty-one extracts from 11 plants are shown, representing different plant parts and extract solvents. For part of plants: Fl.: Flower; Fr.: Fruit; Im.Fr.: Immature Fruit; Lf: Leaf; Rt: Root; Sd: Seed; St.: Stem; W.: Wood; W.St.: Woody Stem. For extract solvents: Aq.: Aqueous; EtOH: Ethanol; MeOH: Methanol. Selectivity index (SI) is also shown. On the lower right side of the panel, growth inhibition of the two most active extracts on *P*. *gingivalis* are shown in relation to tetracycline.

All 21 high-activity extracts were tested for cytotoxic effects on human keratinocytes. Extracts were tested at starting concentrations of 512 μg/mL (4–512 μg/mL) with the exception of *Vicia faba* (MeOH) and *Vitis vinifera* (stem, MeOH), which were tested at starting concentrations of 128 μg/mL (1–128 μg/mL) due to low supply of extract in the QNPL. None of the plant extracts tested had high cytotoxicity; IC_50_ ranged from 256 μg/mL to greater than 512 μg/mL. Sixteen samples had IC_50_ values higher than the range of testing, two (*Azadirachta indica* and *Citrus sinensis*) had IC_50_ values of 512 μg/mL, one (*Sassafras albidum*) had an IC_50_ value of 256 μg/mL, and both samples tested at 128 μg/mL did not exhibit toxicity (Fig 2). When the IC_50_ was undetectable, SI was calculated using the highest concentration of extract tested, and reported as greater than the resulting value. *Sassafras albidum* showed the lowest SI with value of 4, while *Pistacia lentiscus* (fruits, EtOH) showed the highest SI with a value of 256.

## Discussion

### Overview

Of the 416 plant species reported in our literature review, 158 were historically used by North American Native people. To the best of our knowledge, this is the first study to test the activity of plant species historically used by Native Americans for oral health on *P*. *gingivalis*. Fabaceae and Asteraceae were the two most represented botanical families, which has also been found in other studies [55, 56] thus indicating the need to further investigate these families; albeit, this might be due to an overrepresentation of these families as they are the two largest botanical families in the world.

In the screening process, we tested 109 extracts from 21 plant species for their antibacterial activity against *P*. *gingivalis*, of which 21 extracts from 11 plant species showed MICs ranging from 8 to 128 μg/mL. Other studies also tested alcoholic or aqueous plant extracts on the same *P*. *gingivalis* strain (ATCC® 33277) used in our study. For instance, Rosas- Piñón [55] screened 47 plant species from Mexico on *P*. *gingivalis* and four species exhibited the best antibacterial activity with a MIC value of 125 μg/mL. Mohieldin, Muddathir [56] evaluated 24 Sudanese plant species on *P*. *gingivalis*, and the best plant extract had a MIC value of 250 μg/mL. In a study evaluating Japanese herbal medicine, 27 Kampo formulations were tested on *P*. *gingivalis* and the best remedy exhibited an MIC value of 250 μg/mL [57]. In South Africa, eight plant species used traditionally for the treatment of oral diseases were examined, and the best extract showed an MIC value of 800 μg/mL [58]. More generally, an extract with an MIC value less than 100 μg/mL is considered to have significant antibacterial activity [59]. Therefore, of the 21 extracts tested in our investigation, 20 can be considered as having significant antibacterial activity against *P*. *gingivalis*, and our identification of crude extracts with *P*. *gingivalis* MICs as low as 8 μg/mL represents a step forward for the potential of plant-derived treatments for periodontitis.

Aside from the promising antibacterial activity of our extracts, their low cytotoxicity on human cells was also demonstrated. As reported by More et al., a SI greater than 10 (for plant extracts) confirms that the dose that can be administered in most physiological systems [58]. In our study, 13 out of 21 plant extracts had a SI > 10. These results suggest that 13 plant extracts (7 plant species) tested in our study may have therapeutic benefit with acceptable toxicity. However, it is noteworthy that the selectivity index used in this study is based on *in vitro* data from immortalized keratinocytes, and this might not correlate with therapeutic viability in gingival disease [60]. Hereafter, we discuss the potential of the five most promising species as antibacterial agents targeting dental pathogens.

#### Pistacia lentiscus

*Pistacia lentiscus* is an evergreen tree native to the Mediterranean region. The resin from *P*. *lentiscus*, known as mastic, has been used medicinally in traditional medicinal systems widely in the Middle East as well as other areas including Japan, where it is recorded in Kampo medicine. The mastic is chewed as a gum for tooth disease, toothache, or gum inflammation [42, 52]. Crude extract from the fruits of *P*. *lentiscus* had the lowest MIC and highest SI in this study (MIC 8 μg/mL, SI>256).

While the resin has been tested against *P*. *gingivalis*, there were no results in the literature for testing extracts of the fruits. Moreover, the studies focusing on *P*. *lentiscus* resin did not evaluate the MIC, so no direct comparison can be made [61, 62]. Although the chemistry of the fruits has been explored and many compounds identified (e.g., gallic acid, catechin, 3,4-dihydroxyhydro-cinnamic acid, benzoic acid, salicylic acid, and luteolin), it is unknown which compounds in the fruits are responsible for the antibacterial activity observed in this study [63]; given the potent effect of the crude extract, future study should attempt to identify the active constituents of *P*. *lentiscus* fruit.

In the resin of *P*. *lentiscus*, three compounds active against *P*. *gingivalis* have been identified: 24Z-isomasticadienolic acid, oleanolic acid, and oleanonic aldehyde with MIC values of 2.4 ug/mL, 9.8 ug/mL, and 625 ug/mL respectively [64]. These compounds also had growth inhibitory effects against the following oral microbes: *Streptococcus mutans* (24Z-isomasticadienolic acid 78 ug/mL, oleanolic acid 19.5 ug/mL), *Streptococcus sobrinus* (24Z-isomasticadienolic acid 39 ug/mL, oleanolic acid 19.5 ug/mL), *Streptococcus oralis* (24Z-isomasticadienolic acid 39 ug/mL, oleanolic acid 19.5 ug/mL), *Enterococcus faecalis* (24Z-isomasticadienolic acid 156 ug/mL, oleanolic acid 78 ug/mL), and *Parvimonas micra* (24Z-isomasticadienolic acid 2.4 ug/mL) [64].

Aksoy, Duran [65] tested *P*. *lentiscus* mastic gum *in vivo* for antibacterial activity against *Streptococcus* spp. in saliva compared to a placebo gum, finding significantly fewer bacteria in saliva samples after chewing mastic gum. Consistent with the results of our study, no toxicity was detected for 2% *P*. *lentiscus* mastic dissolved in 38% ethanol against keratinocyte cell line (HaCaT), human osteoblastic cell line (Sa-OS-2), mouse fibroblast cell line (Mc3T3-E), and human gingival and periodontal ligament fibroblast cells [61]. Boukeloua et al. [66] examined the *in vivo* toxicity of *P*. *lentiscus* seed oil, finding an LD_50_ of 37 mL/kg for orally ingested seed oil. Smaller doses (100 uL) of orally administered oil daily was found to have no toxic effects on kidneys, livers, or gastrointestinal systems of mice [67]. Oil from the fruits was classified as slightly irritating to the skin and eye of rabbits, but no mortality or organ weight differences were detected [68].

Overall, there is evidence that *P*. *lentiscus* extracts have strong growth inhibitory activity against oral pathogens, low toxicity in several studies, and ability to reduce some oral pathogens *in vivo*. More clinical trials are needed to determine the *in vivo* potential of *P*. *lentiscus* extracts, especially fruit extracts, against *P*. *gingivalis* and periodontitis specifically.

#### Zanthoxylum armatum

*Zanthoxylum armatum*, commonly known as the winged prickly ash, is a deciduous shrub which grows throughout southeast Asia and North America. While it has been used for many medicinal purposes, in India it is used as a chewing stick [37, 69], and the seeds are chewed to cure toothache [70]. In this study, the ethanol extract of *Z*. *armatum* fruits and seeds had an MIC of 16 μg/mL and SI >32.

No previous studies were found testing *Z*. *armatum* extracts against *P*. *gingivalis* or other oral pathogens. Nooreen et al. investigated a methanol extraction of the fruits, and isolated the flavonoids tambulin, prudomestin and ombuin [71]. Ombuin had broad spectrum antimicrobial activity with MIC ranging from 125–500 ug/mL against *Staphylococcus aureus*, *Escherichia coli*, *Pseudomonas aeruginosa*, *Salmonella typhimurium*, *Salmonella typhi*, *Bacillus subtilis*, *Enterococcus faecalis*, *Streptococcus pyogenes*, *Staphylococcus epidermidis* and the oral bacteria *Streptococcus mutans*. However, tambulin had an IC_50_ of 48.7 μg/mL on HaCaT cells.

In another study, 2α-methyl-2β-ethylene-3β-isopropyl-cyclohexan-1β, 3α-diol and phenol-O-β-D-arabinopyranosyl-4′-(3″, 7″, 11″, 15″-tetramethyl)-hexadecan-1″-oate was isolated from the fruits of *Z*. *armatum* and found anti-inflammatory properties *in vitro* [72]. In the same study, macrophages from mice were stimulated by bacterial LPS and production of pro-inflammatory cytokines (TNF-α and IL-6) was significantly inhibited by these compounds.

Overall, very little rigorous research has been done to determine the antibacterial activity of *Z*. *armatum* extracts. Studies must be undertaken to determine the active compounds against *P*. *gingivalis* and other oral pathogens; *in vivo* tests are needed to further understand the toxicity and the potential to reduce oral pathogens in the healthy population and in patients with gingivitis and periodontitis.

#### Juglans regia

*Juglans regia*, commonly known as the Persian Walnut, is a deciduous tree native to Asia and southeastern Europe. In Pakistan and India, the stems and bark have been used for teeth cleaning as chewing sticks [37, 43]. Five extracts of *J*. *regia* were examined in this study, with MIC and SI as follows: immature fruits ethanol extraction (MIC 64 μg/mL, SI >16), immature fruits methanol extraction (MIC 32 μg/mL, SI >32), woody parts ethanol extraction (MIC 32 μg/mL, SI >32), woody stems ethanol extraction (MIC 64 μg/mL, SI >16), and woody stems methanol extraction (MIC 64 μg/mL, SI >16).

Although the plant has not been previously tested against *P*. *gingivalis*, the compound juglone, known to be in *J*. *regia* [73], has activity against *P*. *gingivalis*, with an MIC of 39 μg/mL [74]. However, the similarity of this juglone MIC to the *J*. *regia* crude extract MICs determined in our study (32 to 64 μg/mL) suggests that the anti-*P*. *gingivalis* activity of these complex extracts is not solely due to juglone. No robust studies have specifically investigated *J*. *regia* against oral bacteria with methods following CLSI guidelines [75].

A methanol extract of the bark of *J*. *regia* against 360 strains of 10 multidrug resistant bacterial species [76]. The extract had an MIC of 310 μg/mL against MRSA (methicillin resistant *Staphylococcus aureus*) strains and was selectively active against gram-positive species. In the same study, significant synergy was observed against *Staphylococcus aureus* when *J*. *regia* extract was combined with oxacillin. Chaieb et al. tested an ethanol extract of *J*. *regia* bark against 11 bacterial species for growth inhibition, biofilm inhibition, and biofilm eradication [77]. They found that *J*. *regia* extract had good antibacterial activity against *Listeria monocytogenes*, *Bacillus cereus*, *Staphylococcus epidermidis*, *S*. *aureus*, *Micrococcus luteus* with MIC values ranging from 32 to 64 μg/mL.

Regarding *in vivo* studies, Erdemoglu et al. showed that ethanol extract of the leaves of *J*. *regia* delivered orally to mice has anti-inflammatory effects at 500 mg/kg as determined by the carrageenan-induced paw edema test [78]. When the methanol extracts of the septa of *J*. *regia* was tested for oral acute toxicity and sub-chronic toxicity in rats, no toxicity was detected and the lethal dose was higher than the maximum concentration tested, i.e., 5000 mg/kg [79]

Overall, there is evidence that *J*. *regia* extracts have strong growth inhibitory and antibiofilm activity against many species of bacteria. Juglone, and an active compound from *J*. *regia*, has strong inhibitory activity against *P*. *gingivalis* and other oral pathogens, and extracts of the leaves and septa have low toxicity *in vivo* in mice and rats. More studies are needed to determine the antibacterial activity against a broader range of oral pathogens specifically, and to determine the *in vivo* effects on periodontitis.

#### Citrus sinensis

*Citrus sinensis*, the orange tree, is an evergreen tree of questionable origins most likely in China, northeastern India, or Japan. It has been used as a chewing stick in west Africa [43, 80], and in Malaysia as a decoction made from the leaves for sore mouth [37]. In this study, the methanol extract of woody parts of the plant had an MIC of 32 μg/mL, and SI of 32.

No previous studies were found reporting tests of *C*. *sinensis* leaves or stems against *P*. *gingivalis*. However, extensive antibacterial activity was found for *C*. *sinensis* fruit peel. One study of ethanolic extracts of *C*. *sinensis* peels found limited inhibition of *P*. *gingivalis*, with MICs of 12.5 and 12.8 mg/mL [26], orders of magnitude more than *C*. *sinensis* woody part extract MIC determined in this study, but other studies have demonstrated lower MICs for *C*. *sinensis* peels against other bacteria. For instance, Tao et al. tested the essential oil from the fruit peel with the following results: *Bacillus subtilis* (MIC 9.33 μg/mL), *Staphylococcus aureus* (MIC 4.66 μg/mL) and *Escherichia coli* (MIC 18.75 μg/mL) [81]. Dzotam and Kuete tested methanol extracts of the peel against multidrug resistant gram-negative bacteria and found MICs of 32–512 μg/mL for different *E*. *coli* strains, 128–512 μg/mL for different *Enterobacter aerogenes* strains, 128–512 μg/mL for different *Klebsiella pneumoniae* strains, and 256–512 μg/mL for different *Enterobacter cloacae* strains [82]. The presence of limonene, linalool, citral and myrcene in the essential oil has been linked to its antibacterial activity [83].

Regarding *in vivo* studies, Mandal et al. tested the effects of 4% ethanol extract of *C*. *sinensis* peel mouthwash in patients with moderate to severe gingivitis [84]. The mouthwash showed equivalent efficacy to 0.2% chlorhexidine mouthwash in reducing plaque index, and was more effective than the chlorhexidine mouthwash in reducing gingival inflammation and gingival bleeding.

*C*. *sinensis* extracts present promising antibacterial actions, as well as good *in vivo* activities in a gingivitis model; however, further studies are needed to identify active constituents of and confirm the potential of *C*. *sinensis* stem and leaf extracts as antibacterial agents against oral pathogens.

#### Olea europaea

*Olea europaea*, the olive tree, is an evergreen tree native to southern Europe, the Mediterranean region, and northern Africa. It has been used as a chewing stick for oral hygiene throughout the Middle East [37]. In this study, a methanol extract of the leaves was found to have an MIC of 64 μg/mL and SI >16.

Individual compounds (i.e., hydroxytyrosol, maslinic acid, oleocanthal, oleacein, and oleuropein) from *O*. *europaea* extracts have been tested against *P*. *gingivalis* and other oral pathogens including *Streptococcus mutans*, *Streptococcus sobrinus*, *Streptococcus oralis*, *Fusobacterium nucleatum* and *Parvimonas micra*. Overall, it is likely that maslinic acid and olaecein are responsible for most of the antibacterial activity [64].

Methanol and chloroform extracts of *O*. *europea* leaves had anti-inflammatory effects *in vivo* in the paw edema test in rats [85]. Omer et al. examined *in vivo* toxicity, feeding rats up to 0.9% olive leaf extract in diet for Wistar albino rats for 6 weeks; they found hepatocellular and renal abnormalities, lower cholesterol and blood glucose, and no fatality [86]. Amabeoku et al. tested orally administered methanol leaf extract for acute toxicity in mice, and found an LD_50_ of 3475 mg/kg [87].

Overall, evidence from the literature suggests that compounds from *O*. *europea*, namely olaecein and maslinic acid, have strong growth inhibitory properties against *P*. *gingivalis* and a range of other oral pathogens. The plant extract has anti-inflammatory properties and low toxicity. Further studies are needed to determine whether the plant has any other antibacterial properties against oral pathogens (i.e. biofilm inhibition/eradication), and clinical trials are needed to determine the *in vivo* antibacterial properties in periodontitis.

## Conclusion

In this study, we report for the first time the antibacterial activity of 7 plant species (*Vicia faba*, *Carya alba*, *Juglans regia*, *Citrus sinensis*, *Zanthoxylum armatum*, *Morella cerifera*, *Sassafras albidum*) and one part of *Pistacia lentiscus* (fruits) on *P*. *gingivalis* growth. Each of the 11 plants selected for MIC testing in our study are used in various traditional medical systems in everyday oral hygiene care or as treatments for symptoms related to periodontitis; therefore, the traditional uses of these plants were supported by this study. Moreover, these 11 plants represent a promising collection of sources of natural products which could be further explored for use in pharmaceutical and oral hygiene care product development.

Besides the interesting results found in this work, we also present a method useful for assessing the antibacterial activities of plants against *P*. *gingivalis*. Indeed, *P*. *gingivalis* is an obligate anaerobe which requires specific equipment and skills to cultivate in a lab setting. This partly explains the lack of ethnopharmacological publications focusing on this species. In this work, we developed a rigorous and repeatable methodology for the antibacterial assessment of plant extracts against this pathogen that can be used by scientists for further research.

Future directions based on this research include biofilm testing and bioactivity guided fractionation. When the extracts are fractioned into less complex mixtures, we expect to see even lower MIC values. The extracts should also be tested for growth inhibitory effects on oral commensal species, since some facultative anaerobic bacteria in the oral cavity have important roles in health; for example, they play a role in chemically reducing dietary nitrate [88], and an antibacterial chlorhexidine mouthwash was shown to attenuate this process [89]. Future studies are needed to investigate whether these plant extracts inhibit *P*. *gingivalis* growth while maintaining oral commensals.

One potential product of these results could be development of a mouthwash which includes the tested extracts and/or fractions from these extracts. Because they were found to have high growth inhibitory properties, and *P*. *gingivalis* is a slow-developing bacteria which can take years to start flourishing in the oral cavity, long-term growth inhibition as part of a daily oral hygiene routine could be highly effective.

Periodontal infections affect 47.2% of adults over 30 years old and 70.1% of adults over 65 years old and the development of long-term preventatives could have far-reaching impact. This is especially the case given that *P*. *gingivalis* has been shown to have potential links to so many diseases, including cardiovascular disease, diabetes mellitus, respiratory infection, rheumatoid arthritis, osteoporosis, obesity, pre-term birth and Alzheimer’s disease.

## Supporting information

S1 Fig Impact of vehicle control (DMSO) on A) *P*. *gingivalis* growth and B) human keratinocyte (HaCaT) lysis. The effect of DMSO on *P*. *gingivalis* is displayed as change in optical density during incubation, as described in the methods section, because % inhibition of *P*. *gingivalis* is calculated relative to vehicle control.
(DOCX)

S1 Table Antibacterial screening results of the 109 plant extracts on *Porphyromonas gingivalis* at different concentrations. (DOCX)

## Glossary

AD: Alzheimer’s disease
BHI: Brain Heart Infusion
CFU: Colony Forming Unit
CLSI: Clinical and Laboratory Standards Institute
CNS: Central Nervous System
DMEM: Dulbecco's Modified Eagle Medium
DMSO: Dimethyl sulfoxide
HaCaTs: Human immortalized keratinocytes
IC_50_: Inhibitory Concentration 50%
LDH: Lactate Dehydrogenase
MIC: Minimum Inhibitory Concentration
MRSA: Methicillin resistant *Staphylococcus aureus*
NaOH: Sodium hydroxide
OD: Optical Density
QNPL: Quave Natural Product Library
sBHI: supplemented Brain Heart Infusion
SI: Selectivity Index
