# Supplementary material for: Antibacterial activity of plant species used for oral health against Porphyromonas gingivalis
Source: PLoS One. 2020 Oct 8;15(10):e0239316. doi: 10.1371/journal.pone.0239316 (PMC7544490; doi:10.1371/journal.pone.0239316)
Supplement: S1 Fig — Impact of vehicle control (DMSO) on A) P. gingivalis growth and B) human keratinocyte (HaCaT) lysis. The effect of DMSO on P. gingivalis is displayed as change in optical density during incubation, as described in the methods section, because % inhibition of P. gingivalis is calculated relative to vehicle control. (DOCX) [file pone.0239316.s001.docx]

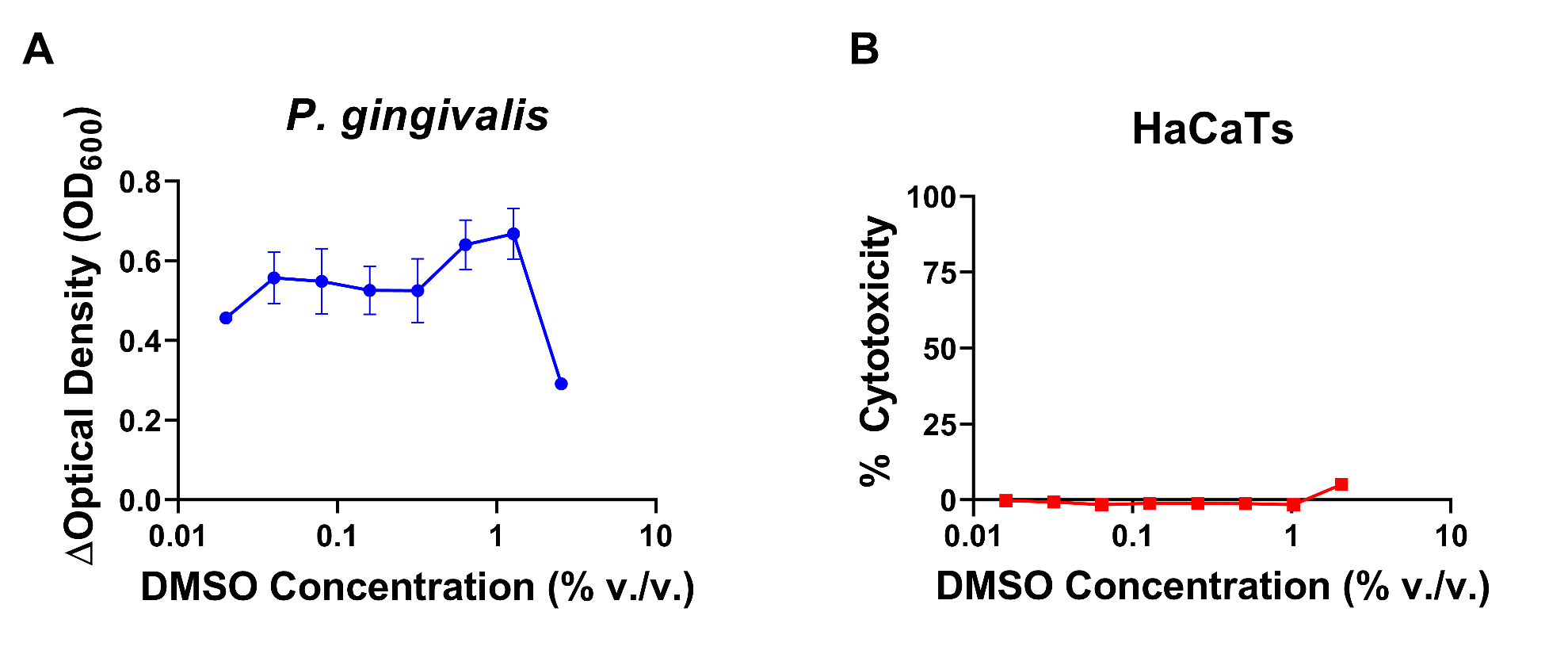
 **S1 Fig:** Impact of vehicle control (DMSO) on A) *P. gingivalis* growth and B) human keratinocyte (HaCaT) lysis. The effect of DMSO on *P. gingivalis* is displayed as change in optical density during incubation, as described in the methods section, because % inhibition of *P. gingivalis* is calculated relative to vehicle control.
