# Supplementary material for: Antibacterial activity of plant species used for oral health against Porphyromonas gingivalis
Source: PLoS One. 2020 Oct 8;15(10):e0239316. doi: 10.1371/journal.pone.0239316 (PMC7544490; doi:10.1371/journal.pone.0239316)
Supplement: S1 Table — (DOCX) [file pone.0239316.s002.docx]

**S1 Table:** Antibacterial screening results of the 109 plant extracts on *Porphyromonas gingivalis* at different concentrations.

| Extract Number | Family | Species | Part | Extract Solvent* | Average % Inhibition (256 μg/mL) | Average % Inhibition (64 μg/mL) | Average % Inhibition (32 μg/mL) |
| --- | --- | --- | --- | --- | --- | --- | --- |
| 634 | Altiginaceae | *Liquidambar styraciflua* L. | woody parts | MeOH | 50.05 ± 3.19 | NT | NT |
| 636 | Altiginaceae | *Liquidambar styraciflua* L. | leaves | MeOH | 90.19 ± 4.38 | — | — |
| 637 | Altiginaceae | *Liquidambar styraciflua* L. | fruits seeds | MeOH | 89.39 ± 8.68 | NT | NT |
| 1637 | Altiginaceae | *Liquidambar styraciflua* L. | roots | 80% EtOH(aq) | 96.6 ± 3.24 | 83.51 ± 2.47 | 19.67 ± 1.54 |
| 1691 | Altiginaceae | *Liquidambar styraciflua* L. | leaves | 80% EtOH(aq) | 69.87 ± 18.57 | NT | NT |
| 1283 | Anacardiaceae | *Pistacia lentiscus* L. | leaves | 95% EtOH(aq) | 91.29 ± 3.14 | 40.92 ± 3.42 | 8.82 ± 7.05 |
| 1288 | Anacardiaceae | *Pistacia lentiscus* L. | leaves | dH_2_O | 98.2 ± 0.6 | 97.36 ± 1.51 | 105.21 ± 6.69 |
| 1300 | Anacardiaceae | *Pistacia lentiscus* L. | woody parts | 95% EtOH(aq) | 102.2 ± 6.07 | 92.55 ± 3.50 | 97.05 ± 2.02 |
| 1457 | Anacardiaceae | *Pistacia lentiscus* L. | woody parts | dH_2_O | 65.17 ± 27.41 | 21.22 ± 14.02 | 11.64 ± 10.54 |
| 1891 | Anacardiaceae | *Pistacia lentiscus* L. | leaves | 95% EtOH(aq) | 78.38 ± 10.39 |  |  |
| 1892 | Anacardiaceae | *Pistacia lentiscus* L. | fruits | 95% EtOH(aq) | 90.09 ± 1.59 | 99.72 ± 0.96 | 98.41 ± 0.88 |
| 142 | Asteraceae | *Achillea millefolium* L. | inflorescence | EtOH | — | NT | NT |
| 153 | Asteraceae | *Achillea millefolium* L. | leaves stems | EtOH | — | NT | NT |
| 159 | Asteraceae | *Achillea millefolium* L. | flowers leaves stems | EtOH | — | NT | NT |
| 234 | Asteraceae | *Achillea millefolium* L. | leaves stems flowers | MeOH | — | NT | NT |
| 235 | Asteraceae | *Achillea millefolium* L. | inflorescence | MeOH | — | NT | NT |
| 646 | Asteraceae | *Achillea millefolium* L. | stems leaves | MeOH | — | NT | NT |
| 656 | Asteraceae | *Achillea millefolium* L. | leaves stems | MeOH | — | NT | NT |
| 1654 | Ebenaceae | *Diospyros virginiana* L. | leaves | 80% EtOH(aq) | 98.3 ± 24.75 | 6.12 ± 3.88 | 4.27 ± 7.14 |
| 1676 | Ebenaceae | *Diospyros virginiana* L. | leaves | 80% EtOH(aq) | 80.58 ± 8.41 | NT | NT |
| 1737 | Ebenaceae | *Diospyros virginiana* L. | stems | 80% EtOH(aq) | 79.38 ± 4.25 | NT | NT |
| 1755 | Ebenaceae | *Diospyros virginiana* L. | immature fruits | 80% EtOH(aq) | 69.37 ± 6.29 | NT | NT |
| 1841 | Ebenaceae | *Diospyros virginiana* L. | woody stems | 80% EtOH(aq) | 72.07 ± 2.35 | NT | NT |
| 66 | Fabaceae | *Vicia faba* L. | leaves flowers whole plant roots stems | EtOH | 97.75 ± 5.31 | 98.12 ± 0.55 | 41.00 ± 10.53 |
| 350 | Fabaceae | *Vicia faba* L. | aerial parts | MeOH | 108.11 ± 2.62 | 98.05 ± 0.24 | 97.76 ± 0.13 |
| 1150 | Fabaceae | *Tamarindus indica* L. | leaves | 95% EtOH(aq) | 43.24 ± 40.79 | NT | NT |
| 619 | Fagaceae | *Quercus alba* L. | bark | MeOH | 79.48 ± 2.72 | NT | NT |
| 620 | Fagaceae | *Quercus alba* L. | galls | MeOH | 87.09 ± 1.31 | NT | NT |
| 632 | Fagaceae | *Quercus alba* L. | leaves | MeOH | 85.49 ± 2 | NT | NT |
| 647 | Fagaceae | *Quercus alba* L. | bark | dH_2_O | — | NT | NT |
| 648 | Fagaceae | *Quercus alba* L. | galls | dH_2_O | 83.93 ± 0.64 | NT | NT |
| 1201 | Fagaceae | *Quercus alba* L. | woody parts | MeOH | 79.18 ± 1.42 | NT | NT |
| 1209 | Fagaceae | *Quercus alba* L. | woody parts | dH_2_O | — | NT | NT |
| 126 | Juglandaceae | *Juglans regia* L. | Immature fruits | EtOH | 91.79 ± 3.04 | 96.17 ± 1.26 | 86.98 ± 2.93 |
| 180 | Juglandaceae | *Juglans regia* L. | leaves | EtOH | 86.19 ± 3.46 | NT | NT |
| 195 | Juglandaceae | *Juglans regia* L. | woody parts | EtOH | 90.19 ± 1.51 | 96.87 ± 0.36 | 97.25 ± 0.13 |
| 275 | Juglandaceae | *Juglans regia* L. | woody parts | MeOH | 71.67 ± 6.04 | NT | NT |
| 276 | Juglandaceae | *Juglans regia* L. | immature fruits | MeOH | 89.89 ± 0.97 | 94.92 ± 1.97 | 94.36 ± 0.57 |
| 277 | Juglandaceae | *Juglans regia* L. | leaves | MeOH | 88.29 ± 3.34 | NT | NT |
| 638 | Juglandaceae | *Carya alba* (L.) Nutt. ex Elliott | woody parts | MeOH | ^†^273.47 ± 17.82 | ^†^131.04 ± 6.17 | 107.09 ± 1.97 |
| 639 | Juglandaceae | *Carya alba* (L.) Nutt. ex Elliott | leaves | MeOH | — | NT | NT |
| 640 | Juglandaceae | *Carya alba* (L.) Nutt. ex Elliott | fruits | MeOH | ^†^359.06 ± 65.04 | ^†^195.76 ± 9.93 | ^†^154.45 ± 11.86 |
| 1511 | Juglandaceae | *Juglans regia* L. | woody stems | 95% EtOH | 96.3 ± 3.31 | 94.99 ± 1.04 | 61.60 ± 9.02 |
| 1512 | Juglandaceae | *Juglans regia* L. | woody stems | MeOH | 92.19 ± 0.6 | 99.93 ± 0.32 | 32.61 ± 7.22 |
| 1582 | Juglandaceae | *Carya alba* (L.) Nutt. ex Elliott | leaves | 80% EtOH(aq) | 89.19 ± 5.24 | 4.38 ± 15.66 | — |
| 1701 | Juglandaceae | *Carya alba* (L.) Nutt. ex Elliott | bark | 80% EtOH(aq) | 81.48 ± 0.92 | NT | NT |
| 1703 | Juglandaceae | *Carya alba* (L.) Nutt. ex Elliott | woody stems | 80% EtOH(aq) | 92.59 ± 8.63 | 36.46 ± 7.85 | 25.60 ± 8.18 |
| 1544 | Lauraceae | *Sassafras albidum* (Nutt.) Nees | leaves | 80% EtOH(aq) | 93.89 ± 0.17 | 12.11 ± 32.59 | 13.16 ± 5.33 |
| 1697 | Lauraceae | *Sassafras albidum* (Nutt.) Nees | roots | 80% EtOH(aq) | 88.69 ± 2 | NT | NT |
| 1795 | Lauraceae | *Sassafras albidum* (Nutt.) Nees | stems | 80% EtOH(aq) | 90.89 ± 1.25 | 99.93 ± 0.24 | 7.52 ± 0.98 |
| 1161 | Meliaceae | *Azadirachta indica* A.Juss. | leaves woody stem | 95% EtOH(aq) | 90.29 ± 1.39 | 96.66 ± 1.37 | 10.85 ± 16.34 |
| 1148 | Moraceae | *Artocarpus altilis* (Parkinson ex F.A.Zorn) Fosberg | leaves | 95% EtOH(aq) | — | NT | NT |
| 747 | Myricaceae | *Morella cerifera* (L.) Small | leaves flowers | MeOH | 25.13 ± 61.23 | 53.5 ± 44.18 | 5.53 ± 2.47 |
| 762 | Myricaceae | *Morella cerifera* (L.) Small | woody parts woody stem | MeOH | 92.79 ± 2.46 | — | — |
| 865 | Myricaceae | *Morella cerifera* (L.) Small | woody stem | dH_2_O | 13.11 ± 6.24 | NT | NT |
| 902 | Myricaceae | *Morella cerifera* (L.) Small | branches stems | MeOH | 85.79 ± 4 | NT | NT |
| 1116 | Myricaceae | *Morella cerifera* (L.) Small | bark | MeOH | 70.97 ± 4.05 | NT | NT |
| 1126 | Myricaceae | *Morella cerifera* (L.) Small | bark | dH_2_O | — | NT | NT |
| 1180 | Myricaceae | *Morella cerifera* (L.) Small | stems, branches | dH_2_O | 6.31 ± 10.23 | NT | NT |
| 132 | Oleaceae | *Olea europaea* L. | leaves | EtOH | 109.51 ± 3.42 | 76.41 ± 2.17 | 26.68 ± 2.85 |
| 293 | Oleaceae | *Olea europaea* L*.* | woody parts | MeOH | 95.6 ± 3.08 | 15.38 ± 1.59 | 12.44 ± 4.23 |
| 294 | Oleaceae | *Olea europaea* L. | leaves | MeOH | 106.61 ± 6.36 | 96.38 ± 1.05 | 36.37 ± 10.77 |
| 122 | Polygonaceae | *Rumex crispus* L. | fruits leaves stems aerial parts | EtOH | 93.19 ± 1.14 | 35.77 ± 5.79 | 17.64 ± 4.93 |
| 378 | Polygonaceae | *Rumex crispus* L. | aerial parts | MeOH | — | NT | NT |
| 402 | Polygonaceae | *Rumex crispus* L. | aerial parts | MeOH | 44.74 ± 25.75 | NT | NT |
| 784 | Polypodiaceae | *Pleopeltis polypodioides* (L.) E.G. Andrews & Windham | whole plant | MeOH | — | NT | NT |
| 786 | Polypodiaceae | *Pleopeltis polypodioides* (L.) E.G. Andrews & Windham | whole plant | dH_2_O | 43.14 ± 5.29 | NT | NT |
| 498 | Rutaceae | *Citrus × sinensis* (L.) Osbeck | fruit rind | MeOH | — | NT | NT |
| 1111 | Rutaceae | *Citrus × sinensis* (L.) Osbeck | woody parts | MeOH | 98.2 ± 0.79 | 96.78 ± 0.32 | 94.79 ± 0.57 |
| 1236 | Rutaceae | *Citrus × sinensis* (L.) Osbeck | woody parts | MeoH | — | NT | NT |
| 1290 | Rutaceae | *Zanthoxylum armatum* DC. | fruits, seeds | 95% EtOH(aq) | 117.12 ± 1.97 | 98.82 ± 0.84 | 96.6 ± 0.33 |
| 774 | Salicaceae | *Salix nigra* Marshall | leaves | MeOH | — | NT | NT |
| 897 | Salicaceae | *Salix nigra* Marshall | leaves | dH_2_O | 54.65 ± 14.25 | NT | NT |
| 922 | Salicaceae | *Salix nigra* Marshall | flowers fruits leaves | MeOH | 73.37 ± 6.59 | NT | NT |
| 1023 | Salicaceae | *Salix nigra* Marshall | branches | MeOH | 64.46 ± 6.69 | NT | NT |
| 1028 | Salicaceae | *Salix nigra* Marshall | woody stem | MeOH | 91.09 ± 2.5 | 27.14 ± 18.01 | — |
| 1030 | Salicaceae | *Salix nigra* Marshall | woody stem | dH_2_O | 87.99 ± 4.92 | NT | NT |
| 1031 | Salicaceae | *Salix nigra* Marshall | branches | dH_2_O | — | NT | NT |
| 1065 | Salicaceae | *Salix nigra* Marshall | leaves fruits flowers | dH_2_O | — | NT | NT |
| 1071 | Salicaceae | *Salix nigra* Marshall | woody stem | MeOH | — | NT | NT |
| 1073 | Salicaceae | *Salix nigra* Marshall | leaves | MeOH | 86.29 ± 2.33 | NT | NT |
| 1134 | Salicaceae | *Salix nigra* Marshall | bark | MeOH | 85.49 ± 2.27 | NT | NT |
| 1136 | Salicaceae | *Salix nigra* Marshall | bark | MeOH | 82.78 ± 4.03 | NT | NT |
| 1185 | Salicaceae | *Salix nigra* Marshall | bark | MeOH | — | NT | NT |
| 1206 | Salicaceae | *Salix nigra* Marshall | bark | dH_2_O | 85.79 ± 8.11 | NT | NT |
| 1210 | Salicaceae | *Salix nigra* Marshall | woody stems | dH_2_O | 96.55 ± 0.21 | 15.80 ± 2.95 | 15.26 ± 11.56 |
| 1211 | Salicaceae | *Salix nigra* Marshall | bark | dH_2_O | 97.5 ± 0.76 | 16.77 ± 5.39 | 14.53 ± 3.96 |
| 1576 | Salicaceae | *Salix nigra* Marshall | leaves | 80% EtOH(aq) | 91.39 ± 3.8 | — | — |
| 1749 | Salicaceae | *Salix nigra* Marshall | bark | 80% EtOH(aq) | 84.38 ± 4.26 | NT | NT |
| 1783 | Salicaceae | *Salix nigra* Marshall | roots | 80% EtOH(aq) | 105.01 ± 9.82 | 26.23 ± 0.73 | 3.47 ± 15.98 |
| 919 | Sapotaceae | *Sideroxylon celastrinum* (Kunth) T.D. Penn. | stems | MeOH | 16.22 ± 72.77 | 81.14 ± 3.56 | 25.52 ± 6.95 |
| 1182 | Sapotaceae | *Sideroxylon celastrinum* (Kunth) T.D. Penn. | leaves, stems | MeOH | 73.97 ± 9.99 | NT | NT |
| 1190 | Sapotaceae | *Sideroxylon celastrinum* (Kunth) T.D. Penn. | leaves, stems | dH_2_O | — | NT | NT |
| 1225 | Sapotaceae | *Sideroxylon celastrinum* (Kunth) T.D. Penn. | stems | dH_2_O | — | NT | NT |
| 1609 | Sapotaceae | *Sideroxylon lanuginosum* Michx. | leaves | 80% EtOH(aq) | 80.18 ± 6.5 | NT | NT |
| 1718 | Sapotaceae | *Sideroxylon lanuginosum* Michx. | leaves | 80% EtOH(aq) | 74.97 ± 3.32 | NT | NT |
| 1756 | Sapotaceae | *Sideroxylon lanuginosum* Michx. | woody stems | 80% EtOH(aq) | 85.69 ± 1.14 | NT | NT |
| 1864 | Sapotaceae | *Sideroxylon lanuginosum* Michx. | bark | 80% EtOH(aq) | 94.19 ± 1.42 | 43.70 ± 5.25 | 30.59 ± 4.34 |
| 120 | Vitaceae | *Vitis vinifera* L. var. *aglianico* | stems | EtOH | 87.69 ± 6.82 | NT | NT |
| 123 | Vitaceae | *Vitis vinifera* L. var. *aglianico* | fruits | EtOH | 92.69 ± 2.27 | 14.96 ± 10.63 | 10.48 ± 14.91 |
| 124 | Vitaceae | *Vitis vinifera* L. var. *aglianico* | leaves | EtOH | 93.99 ± 2.97 | 94.05 ± 0.15 | 6.22 ± 1.48 |
| 335 | Vitaceae | *Vitis vinifera* L. var. *aglianico* | fruits | MeOH | — | NT | NT |
| 336 | Vitaceae | *Vitis vinifera* L. var. *aglianico* | leaves | MeOH | 98.1 ± 6.13 | 97.22 ± 0.48 | — |
| 337 | Vitaceae | *Vitis vinifera* L. var. *aglianico* | stems | MeOH | 136.04 ± 14.59 | 100.77 ± 0.43 | 97.98 ± 0.63 |
| 481 | Vitaceae | *Vitis rotundifolia* Michx. | leaves stems | MeOH | 81.83 ± 1.49 | NT | NT |
| 1613 | Vitaceae | *Vitis rotundifolia* Michx. | leaves | 80% EtOH(aq) | 81.78 ± 10 | NT | NT |
| 1760 | Vitaceae | *Vitis rotundifolia* Michx. | roots | 80% EtOH(aq) | 85.59 ± 6 | NT | NT |
| 1831 | Vitaceae | *Vitis rotundifolia* Michx. | woody stems | 80% EtOH(aq) | 67.07 ± 64.46 | 24.91 ± 3.79 | — |
| 1837 | Vitaceae | *Vitis rotundifolia* Michx. | immature fruits | 80% EtOH(aq) | 80.48 ± 0.79 | NT | NT |

* dH_2_O: Distilled water. EtOH: Ethanol. EtOH(aq): aqueous ethanol. MeOH: methanol. “-“ signifies not active at the test concentration. “NT”: not tested. ^†^ Optical density readings impacted by clumping of bacterial cells in test wells (issue for tannin-rich extracts).
